# Persistent problems of access to appropriate, affordable TB services in rural China: experiences of different socio-economic groups

**DOI:** 10.1186/1471-2458-7-19

**Published:** 2007-02-08

**Authors:** Tuohong Zhang, Shenglan Tang, Gao Jun, Margaret Whitehead

**Affiliations:** 1 Department of Health Policy and Management, School of Public Health, Peking University, 38 Xueyuan Road, Beijing 100083, P. R. China; 2 International Health Group, Liverpool School of Tropical Medicine, Pembroke Place, Liverpool, L3 5QA, UK; 3 The Center for Health Statistics and Information (CHSI), Ministry of Health, Beijing, P.R China; 4 Division of Public Health, School of Population, Community and Behavioural Sciences, Whelan Building, Quadrangle, The University of Liverpool, Liverpool, L69 3GB, UK

## Abstract

**Background:**

Large-scale Tuberculosis (TB) control programmes in China have been hailed a success. Concerns remain, however, about whether the programme is reaching all sections of the population, particularly poorer groups within rural communities, and whether there are hidden costs. This study takes a household perspective to investigate receipt of appropriate care and affordability of services for different socio-economic groups with TB symptoms in rural China.

**Methods:**

Secondary analysis of Chinese National Household Health Survey for 2003: 40,000 rural households containing 143,991 individuals, 2,308 identified as TB suspects. Outcomes: use of services and expenditure of TB suspects, by gender and socio-economic position, indicated by household income, education, material assets, and insurance status.

**Results:**

37% of TB suspects did not seek any professional care, with low-income groups less likely to seek care than more affluent counterparts. Of those seeking care, only 35% received any of the recommended diagnostic tests. Of the 182 patients with a confirmed TB diagnosis, 104 (57%) received treatment at the recommended level, less likely if lacking health insurance or material assets. The burden of payment for services amounted to 45% of annual household income for the low-income group, 16% for the high-income group.

**Conclusion:**

Access to appropriate, affordable TB services is still problematic in some rural areas of China, and receipt of care and affordability declines with declining socio-economic position. These findings highlight the current shortcomings of the national TB control programme in China and the formidable challenge it faces if it is to reach all sections of the population, including the poor with the highest burden of disease.

## Background

High prevalence of tuberculosis (TB) is a critical public health problem in China. By the year 2000, there were an estimated 4.7 million prevalent cases of TB across the country, 1.6 million of whom were sputum smear-positive. Each year, about 1.4 million new TB cases and 150,000 TB-related deaths are recorded [1].

Over the past decade, the seriousness of the situation has led to the introduction by the Chinese Ministry of Health of large-scale TB control programmes, with support from the World Bank. These programmes, employing the WHO-recommended Directly Observed Therapy Strategy (DOTS), have met with considerable success in the places in which they have been introduced, as reported in *The Lancet *in 2004 [1]. Dye and colleagues compared the trends from 1990 to 2000 in prevalence of TB in the 13 provinces that had introduced the DOTS programme with 15 non-programme provinces. Prevalence fell by 48% in the DOTS provinces compared with 16 percent in the provinces without the programme, resulting in an estimated 30 percent greater reduction in TB prevalence, which the authors attributed directly to the DOTS programme.

These falls in prevalence certainly indicate remarkable achievements in TB control in China overall. Nevertheless, questions remain about the relative contribution of the DOTS programme versus concomitant changes in socio-economic factors [2], as well as concerns that a substantial number of patients are not getting the best quality treatment available in the official TB dispensary system. Dye and colleagues, for example, found that of the pulmonary TB patients who had been diagnosed before the 2000 survey, only 12 percent received their diagnosis at TB dispensaries and only 13 percent diagnosed elsewhere were eventually transferred to the dispensaries [1]. Given that treatment in the TB dispensary system is supposedly free and of higher quality as measured by achieved cure and transmission rates, why are the majority of diagnosed TB patients getting poorer-quality care elsewhere? Are some TB suspects receiving no care at all? What is the role of patient's financial circumstances?

Such questions need to be addressed to suggest ways to improve implementation and coverage of the most effective control programmes for TB. In China, evidence on health care access for different socio-economic groups is extremely scarce, though some are now beginning to examine this question. In 2002, an institution-based survey of 493 newly diagnosed TB patients in two relatively prosperous rural counties (one with and one without a National TB Control Programme (DOTS programme)), found considerable diagnostic delays in access to TB care in both counties, with, surprisingly, a longer total delay in the DOTS programme covered county. Less educated and uninsured patients suffered longer delays in the DOTS county, while poor patients and farmers suffered longer delays in the non-DOTS county [3]. In a 2004 survey of 20 villages in one Chinese county, 171 patients with long-term chronic cough (potential TB cases) were identified from the total population and interviewed. Of the total of 171, only 115 had ever seen a doctor, and only 11 of these had received a smear sputum test [4]. Uninsured patients, farmers and those living far from facilities had longer delays to contact with first health provider.

Prior to the 2003 National Household Health Survey, however, there was no nationally representative study examining socio-economic differences in utilization of health services by members of the general population in need of TB care. This national survey provides individual information about symptoms of long-lasting cough, together with data on socio-economic status, health-seeking behaviour, and TB-related diagnosis and treatment. This provided us with the opportunity to explore how the experiences of people from different socio-economic groups with symptoms of TB differ along the pathways to effective care. Specifically, this paper reports analyses that ask: Who gets care and who does not? What is the burden of payment for medical care for TB suspects and patients and how does it differ by socio-economic status?

## Methods

### Data source

Data from the National Household Health Survey (NHHS) in China conducted in 2003 were analysed [5]. This survey was commissioned and carried out under the supervision of the Centre for Health Statistics and Information of the Chinese Ministry of Health, overseen by one of the authors (GJ). A four-stage stratified random sampling procedure was used to select households for interview, to be representative of both the urban and rural population of China. In the analysis reported in this paper, we used only the rural component of the dataset. There were 67 rural counties selected, in which 40,600 households were randomly selected for interview. Of these, a total of 40,212 rural households agreed to participate, containing 143,991 individuals [5]. Two enumerators visited the selected households to conduct face-to-face interviews with all family members one by one. All adults were asked to answer the questions for themselves, however, 20 percent of adults were not at home because of seasonal migrant work, in which case the head of the household answered on their behalf. Mothers usually answered questions for their children who were younger than 15 years old. Questions on education and occupation were not applicable to children under 15, leading to some missing values for some variables (see footnotes for Table 1).

**Table 1:** Number and prevalence of TB suspects by socio-demographic characteristics in rural China, 2003

*Socio-demographic factors*	*No. of population*	*Number of TB suspects*	*Prevalence (/100,000)*	*X*^2^	*P Value*
	(1)	(2)			

**Age group**				2263.502	<0.0001
<30	62400	235	376		
30–44	36062	425	1178		
45–59	28914	772	2669		
60 & over	16614	876	5272		
**Sex**				3.082	0.079
female	70556	1082	1533		
Male	73435	1226	1669		
**Health insurance**				30.727	<0.0001
No	129303	2005	1550		
Yes	13630	297	2179		
**Education**				689.508	<0.0001
Illiterate	25369	932	3673		
Primary school	34623	774	2235		
Secondary school and above	51095	470	919		
**Occupation**				33.696	<0.0001
Farmer	80317	1692	2106		
Non-farmer	30808	483	1567		
**Household income**				24.202	<0.0001
Low	46657	854	1830		
Medium	48177	691	1434		
high	48604	763	1569		
**Colour TV holding**				109.610	<0.0001
No	58804	1188	2020		
Yes	85089	1119	1315		
Total No. of population	143,991	2308	1602		

Note:

(1) Some cases with missing value for total population were taken as missing cases: 1 missed for age group;1058 missed for health insurance; 32904 missed and unreported for education; 32866 missed and unreported for occupation; 553 missed for household income; 98 missed for colour TV holding.

(2)Some cases with missing value for suspects were taken as missing cases: 6 missed for health insurance; 131 missed and unreported for education and occupation; 2 missed for household colour TV holding.

A number of measures on quality assurance were taken during the period of data collection. Five percent of the households sampled were re-visited by the supervisors to double check that the information recorded by the first numerator was correctly taken down in the questionnaire. The rates of consistency in recording between the numerators and the supervisors were about 97% for major indicators. A logic check of all the data collected was also undertaken to examine if there were any contradictions or information that did not make any sense. Triangulation with data from the routine health information system found that estimations of health facility use at a certain level, such as the township level, by the household survey were comparable with estimations of use at the same level by routine data. This finding together with other tests, has convinced us that the study population sampled was representative.

### Socio-economic and heath related variables

The socio-demographic variables used were:

• Age (four groups)

• Sex

• Health insurance coverage (yes or no)

• Education (three groups: illiterate; primary school only; secondary school and above)

• Occupation of head of household in rural areas (two groups: farmer or not farmer)

• Household assets (two groups: owning colour television or not).

• Household income (three groups: low, medium and high). These groups were formed by ranking individuals by their income and then dividing the ranking into three categories, each containing a third of the total number of households. As there is no practical way of separating individual from household income in rural China, estimates of individual income were made for this analysis by taking the reported gross household income and dividing it by the number of individuals in the household.

### Variables related to health and care-related factors

Two of the authors (ST and GJ) were responsible for the development of 13 TB-related questions, which were inserted into the 2003 NHHS. Interviewees who would be considered medically to be in need of professional advice on TB care were identified by the following question:

#### TB suspects

*Self-reported cough of more than 3 weeks duration during the 12 months preceding interview*. Although a long lasting cough is clinically very suggestive of TB, in the context of rural China it is also a common symptom for other respiratory diseases, such as asthma and chronic bronchitis. We therefore refer to the people reporting a persistent cough with this question as "TB suspects" in this paper.

***Health care utilization **related to a cough of more than 3 weeks duration *including:

• consultation with a medical doctor in the previous 12 months;

• having undergone chest radiography;

• having had a sputum smear test;

• having been treated at a county TB dispensary/CDC or a general hospital;

• cost incurred for medical treatment over the previous 12 months.

### Statistical analysis

Prevalence of people with TB symptoms was calculated for each age and socio-economic group per 100,000 population. Age-adjusted odds ratios (with Wald 95% confidence intervals) were calculated by running logistic regression models according to the methods of Armitage and Colton [6]. These odds ratios estimated the chances of different socio-economic groups gaining access to specified types of care compared to the chances for the most privileged group in each category, adjusted for the differing age structure of the socio-economic groups.

### Analytical framework

The framework developed by the Affordability Ladder Program (ALPS) was employed to structure the analysis [7]. This approach is based on a household perspective. Starting with individuals in households who have a health problem/condition, it then examines in a systematic way the pathways to the effective care that these individuals need for their particular health problem and how the actual experiences of gaining access to care and its affordability differ for different socio-economic groups in the population.

Main elements of the approach include examining the following questions:

1. Who did not get any care for their problem? Why did they not seek care and what were the consequences?

2. Who received informal care only? Was this appropriate for this health problem?

3. Who gained access to and utilisation of professional care? How did the pattern of use of professional care vary by socio-economic status?

4. Did the quality of professional care received vary (in terms of medical quality, skill, attitudes of medical staff, safety and waiting time) and if so how?

5. Burden of payment for health services and drugs: what did different types of household have to pay for their professional care and medications? How big a burden was this payment on the household budget and how did that differ by socio-economic groups?

The presentation of results follows this step-wise framework

## Results

### Number of TB suspects

From the 2003 national survey, 2,308 rural residents out of 143,991 individuals in the rural sample reported a persistent cough of more than 3 weeks during the 12 months before interview, giving a crude prevalence of 1603 TB suspects per 100,000 rural population. The age-specific rates increased with age, and prevalence was higher among farmers than among non-farmers, among those with lower education level than those with higher education level, and among the low-income group than among the high income groups (Table 1). All these differences were statistically significant. Male rural residents had a higher rate than their female counterparts, but the difference was not statistically significant.

### Which TB suspects did not seek care for their problem?

Among 2,308 TB suspects, 1,452 (63%) had visited a doctor at least once during the 12 months before the survey, while over a third (37%) did not seek any professional care for their symptoms (Table 2). Proportions not seeing a doctor were higher for men than for women; in low-income households than in higher income ones; and for households not owning a colour TV than for ones with a colour TV. All these differences were statistically significant. Age, health insurance status, education and occupation were not statistically significant factors here.

**Table 2:** Percentage of TB suspects not seeking professional care, by socio-demographic characteristics, rural China, 2003

Influencing factors	No. TB suspects	No. TB suspects who did not go to see a doctor	% TB suspects who did not go to see a doctor	X^2^	P value
Total	2308	856	37.1		
**Age group**					
<30	235	72	30.6	7.054	0.070
30–44	425	173	40.7		
45–59	772	293	38.0		
60 & over	876	318	36.3		
**Sex**					
female	1082	378	34.9	4.047	0.044
Male	1226	478	39.0		
**Health insurance**					
No	2005	752	37.5	1.358	0.244
Yes	297	101	34.0		
**Education**					
Illiterate	932	352	37.8	4.304	0.116
Primary school	774	309	39.9		
Secondary school and above	470	160	34.0		
**Occupation**					
Farmer	1692	638	37.7	0.005	0.942
Non-farmer	483	183	37.9		
**Household income**					
Low	854	342	40.0	7.648	0.022
Medium	691	259	37.5		
High	763	255	33.4		
**Colour TV holding**					
No	1188	467	39.3	5.104	0.024
Yes	1119	389	34.8		

Note:

Some cases with missing value for suspects were taken as missing cases: 6 missed for health insurance; 131 missed and unreported for education and occupation; 2 missed for household colour TV holding.

Table 3 presents comparisons across income, education and insurance groups. The odds ratios indicate the chances of TB suspects from different socio-economic groups consulting a doctor for their symptoms compared with the most advantaged category. After age adjustment, male and female TB suspects with low income were less likely to go to see a doctor than their high income counterparts (OR 0.75, CI 0.56 – 0.99; and OR 0.74, CI 0.54 – 0.99 respectively). The illiterate male TB suspects and those with only primary education were over 40 percent less likely to see a doctor for their TB symptoms than those with secondary education or higher (OR 0.60, CI 0.42 – 0.85; and OR 0.62 CI 0.46 – 0.84 respectively). There was no difference in seeking care among female TB suspects from different educational groups, or for those with or without health insurance.

**Table 3:** Age-adjusted odds ratios (with 95% confidence intervals) for TB suspects seeking professional care by sex and socioeconomic group, rural China, 2003

	Male		Female	
	
	OR	95%CI	OR	95%CI
**Income**				
Low	0.75	(0.56 – 0.99)	0.74	(0.54 – 0.99)
Medium	0.82	(0.61 – 1.10)	0.87	(0.63 – 1.21)
High	1.00		1.00	

**Education**				
Illiterate	0.60	(0.42 – 0.85)	0.80	(0.51 – 1.26)
Primary	0.62	(0.46 – 0.84)	0.81	(0.53 – 1.25)
Secondary	1.00		1.00	

**Health insurance**				
No	0.93	(0.65 – 1.33)	0.78	(0.54 – 1.13)
Yes	1.00		1.00	

### Uptake of diagnostic tests

Among TB suspects who did seek professional care, only a minority received diagnostic X-ray examination for their symptoms: 629 (27%) of the total number of identified TB suspects. Table 4 shows that both male and female TB suspects with low income were less likely to have an x-ray examination for their TB symptoms than those with high income (male: OR = 0.73, 95% CI 0.55–0.99; female: OR = 0.63, 95% CI 0.45 – 0.89). In addition, male TB suspects without educational qualification or with only primary school education, were less likely to have an X-ray examination (Illiterate: OR = 0.54, 95% CI 0.37–0.78; Primary school: OR = 0.63, 95% CI 0.46–0.87).

**Table 4:** Age-adjusted odds ratios (95% confidence intervals) for TB suspects given X-ray examination by sex and socioeconomic group, rural China, 2003

	Male		Female	
	
	OR	95%CI	OR	95%CI
**Income**				
Low	0.73	(0.55 – 0.99)	0.63	(0.45 – 0.89)
Medium	0.75	(0.55 – 1.03)	0.93	(0.66 – 1.31)
High	1.00		1.00	

**Education**				
Illiterate	0.54	(0.37 – 0.78)	1.12	(0.68 – 1.86)
Primary	0.63	(0.46 – 0.87)	1.11	(0.69 – 1.81)
Secondary	1.00		1.00	

**Health insurance**				
No	0.89	(0.61 – 1.30)	0.98	(0.66 – 1.46)
Yes	1.00		1.00	

Using a sputum test to diagnose TB cases is one of five elements in the DOTS programme. In China, where there is a high DOTS coverage, the use of a sputum test for TB diagnosis should be widely implemented, but our results indicate that it was not so. Our study found that only 216 (9.4%) of the TB suspects received a sputum test. Table 5 gives some indication of increasing odds of having a sputum test with decreasing income for both men and women, and with decreasing education in men, but the confidence intervals include 1.00, so the differences were not statistically significant. Male TB suspects without health insurance, however, were statistically less likely to have a sputum test than those covered by health insurance (OR = 0.58, 95% CI 0.35 – 0.94).

**Table 5:** Age-adjusted odds ratios (95% confidence intervals) for TB suspects given sputum test by sex and socioeconomic group, rural China, 2003

	Male		Female	
	
	OR	95%CI	OR	95%CI
**Income**				
Low	1.52	(0.96 – 2.39)	1.50	(0.88 – 2.55)
Medium	1.17	(0.71 – 1.92)	1.31	(0.74 – 2.32)
High	1.00		1.00	

**Education**				
Illiterate	1.37	(0.78 – 2.40)	1.23	(0.57 – 2.64)
Primary	1.41	(0.87 – 2.28)	0.67	(0.31 – 1.45)
Secondary	1.00		1.00	

**Health insurance**				
No	0.58	(0.35 – 0.94)	0.85	(0.47 – 1.55)
Yes	1.00		1.00	

### Dropout of TB suspects along the pathways to treatment

**Figure 1:**
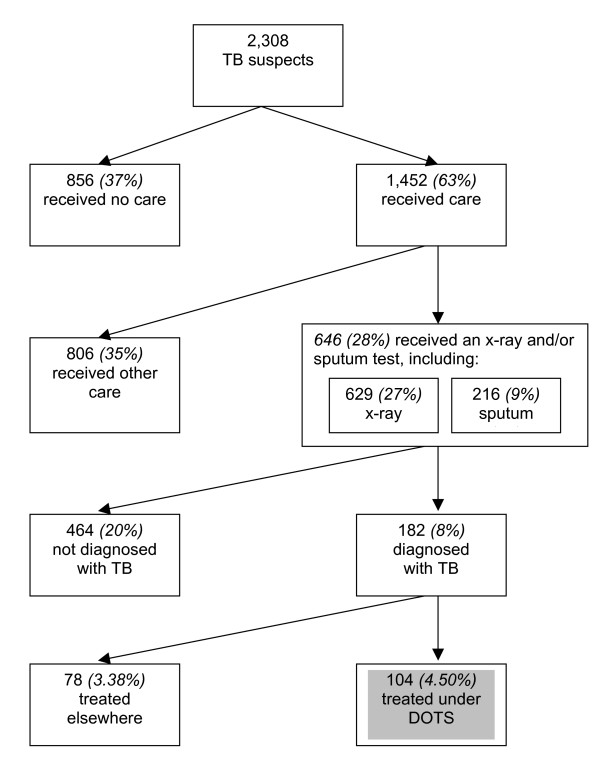
**Dropout of TB suspects from the health care system in rural China, 2003**. Numbers in italics show percentage of the original sample of 2308 TB suspects who reach this stage of care. There were 199 patients who received both the x-ray examination and the sputum test.

Figure 1 illustrates the considerable dropout at each stage in the pathways from TB symptom to diagnosis and treatment. It is not possible from the data to identify how many of the 856 TB suspects who received no care at all actually needed professional care, and how many had a mild problem that did not require further attention.

Given the recommendations of the DOTS programme, which covered 91 percent of the population by the end of 2003, it could be argued that a vast majority of the 1452 (63%) TB suspects who did see a doctor for their symptoms should have received diagnostic tests for TB. From our study, 806 cases (35%) did not receive any recommended diagnostic tests. Only 216 TB suspects (9%) received the recommended sputum test, while 629 (27%) of TB suspects seeking care were given an X-ray examination.

Of 182 patients who had a confirmed diagnosis of TB, the DOTS protocol suggests that they should be treated at the county TB dispensary level. In reality, only 104 (just over a half) of diagnosed patients, were treated at the county TB dispensaries or CDC, which represented only 4.5 percent of the original sample of TB suspects. The proportion treated under DOTS was much smaller among the patients without insurance and among those whose households did not have materials assets such as colour TVs.

### Burden of payment for TB care among different socio-economic groups

To explore the role of economic factors in this pattern of dropout, we estimated medical expenses for diagnosis and treatment of TB suspects and registered TB patients, by using a proxy indicator: annual household medical expenses for all medical conditions over the last 12 months. This proxy was available at the level of individual cost data, whereas TB-specific expenditure was not. We justified the use of this indicator because the treatment course for TB lasted for at least 6 months, often up to 8 months, and could thus constitute a substantial proportion of the overall burden of payment over the period. It could also be considered as an indicator of the overall financial burden due to medical expenses faced by the households containing TB suspects and patients.

Low-income families with diagnosed TB patients or suspects were in extremely bad financial circumstances (Table 6). For the poor households with TB suspects, total annual income was 2718 Yuan, whilst their annual household expenses were 4158 Yuan, a 53 percent shortfall in income. The poor families with TB patients suffered a similar situation. In the poor families with either TB suspects or patients, more than 40 percent of the total annual household income was taken up with medical expenses. Even for middle and high income groups, medical expenses took up a substantial proportion of household income: 19.3 percent and 16.7 per cent respectively. The burden of payment for medical care resulted in 60 percent of the households with TB suspects or patients having to borrow money, and 9 percent had to give up the treatment.

**Table 6:** Annual household medical costs as percentage of annual household expenditure and annual household income for TB suspects and diagnosed TB patients in 2002, by income group

	Total household medical expense (Yuan)	Total household income (Yuan)	Total household expenditure (Yuan)	Medical expense as % of total household income = /	Medical expense as % of total household expenditure = /
**TB suspects (N = 2308)**					
Low income group (N = 854)	1239	2718	4158	45.6	29.8
Middle income group (N = 691)	1121	5810	5835	19.3	19.2
High income group (N = 763)	2147	12835	9946	16.7	21.6
**TB patients (N = 180)**					
Low income group (N = 80)	1241	2954	4105	42.0	30.2
Middle income group (N = 56)	2061	7393	8033	27.9	25.7
High income group (N = 46)	2090	11219	9198	18.6	22.7

To take some account of possible confounding by age and co-morbidity, we also compared the per capita expenditure patterns of the households with TB suspects and registered TB patients with those of the general population, across income groups, for the age group of 45 to 59 years old (Table 7). There was a clear gradient in medical expenses, with TB patients paying the highest proportion of their per capita annual income on medical expenses, followed by TB suspects, with the general population paying the lowest proportion. A similar gradient was evident in each of the three income groups, though the impact of the medical expenses on the household income was the greatest in the low-income group. This resulted in the payment of medical expenses by TB patient households equivalent to 112 percent of the low-income households' per capita annual income, 19.9 percent for the middle-income households and 32.7 percent for the high-income households.

**Table 7:** Per capita medical costs as percentage of per capita expenditure and per capita income for the general population, TB suspects and diagnosed TB patients aged 45–59 years in 2002, by income group

	Per capita medical expense	Per capita income	Per capita expenditure	Medical expense as % of income = /	Medical expense as % of expenditure = /
**Low-income group**					
General population(N = 7491)	213	766	1053	27.7	20.2
TB Suspects(N = 238)	329	718	1162	45.8	28.3
TB Patients(N = 25)	823	735	1626	112.0	50.6
**Middle-income group**					
General population(N = 9184)	215	1624	1543	13.2	13.9
TB Suspects(N = 223)	290	1616	1698	17.9	17.1
TB Patients(N = 21)	317	1590	1451	19.9	21.8
**High-income group**					
General population(N = 12239)	386	4371	3077	8.8	12.5
TB Suspects(N = 311)	798	4200	3394	19.0	23.5
TB Patients(N = 16)	1081	3370	2984	32.7	36.2

## Discussion

Concerted action is needed to control the TB epidemic and reduce prevalence in China in the next decade, involving easy access to affordable TB care and an effective implementation of DOTS programme, particularly in the poor rural areas. In other words, equity in use and finance of TB related services and quality of DOTS programme implementation hold a key to future success of TB control in China, which has been facing many problems and challenges [8], albeit being relatively successful [1,9].

### Strengths and limitations of the study

The data for this study come from the Chinese National Household Health Survey, which has both strengths and limitations. A major strength is that the sample covers the whole of rural China, and is nationally representative, unlike other studies that have focused on one or only a few counties. The sample size is large, giving the analyses good statistical power. In addition, because the sample was drawn from the population at large, not just those in contact with health services, it includes individuals who have a health problem but who have not used medical care for one reason or another. This is an advantage over studies that recruit participants from facilities and thus exclude those who do not get a professional diagnosis, which are likely to be the poorer socio-economic groups.

A number of limitations need to be borne in mind. First, the survey relied on self-reports of health problems, including TB symptoms and diagnoses: there were no clinical measurements made in the survey. On the one hand this means that some TB cases may be missed that would have been picked up with diagnostic tests. On the other hand, the question asked in the survey about "self-reported cough of more than three weeks duration" will include individuals who do not have TB but have some other respiratory problem. We acknowledge this limitation by referring to the individuals who answer "yes" to this problem as "TB suspects". The legitimacy for using this working definition derives from the fact that this symptom of a cough of more than three weeks duration indicates, at the very least, a need for medical advice. We thus use it as a tracer condition, to trace the pathways to health care of people in different socio-economic groups who should get a medical consultation and tests for TB. There is less ambiguity about the status of TB patients in the study. The 2003 survey had the advantage over previous ones of including 13 specific questions relating to TB, including ones on confirmed diagnoses. A total of 182 people reported that they had a doctor-confirmed diagnosis of TB and their medical expenditure was analysed accordingly. As TB is widely recognised as a serious condition that has a considerable impact on a sufferer's daily life, we have no reason to doubt the accuracy of these self-reported diagnoses.

A second area of limitation in the study is the use of total medical expenditure in tables 6 and 7, rather than purely TB expenditure data, because the survey questionnaire did not allow this differentiation. The total annual household medical expenditures could include considerable expenditure on obstetric care, cancer or other expenditures unrelated to TB. Although we acknowledge that it would have been preferable to have TB expenditure, we argue that the available data on total medical expenditure is still worth analysing. It provides valuable information on the financial burden of paying for medical care on different types of household containing a TB suspect or patient.

### Equity in access to TB care and financial burden of care

Equity in access to health services remains a serious issue in many developing countries including China. Our study found that over one third of TB suspects did not seek any professional care after a persistent cough for more than 3 weeks. Those with low income were less likely to seek professional care than their rich counterparts. Whether or not people seek professional care when they are ill may be affected by many factors. Gao and colleagues [10] found that the urban Chinese population were more likely to undertake self-care than previously, and less likely to seek professional care when they became ill, owing both to the rapid rise of medical care expenses and to the decline in the number of people covered by insurance. In rural China, there is an added problem of adequacy of available insurance. The rural CMS does not provide a good benefit package for the subscribers and in addition the insurance schemes often require high co-payments. Given these deficiencies, therefore, it is not surprising that in our study there was no statistically significant difference between the insured and uninsured groups in the proportion not seeking any professional care (Table 2).

According to clinical advice, individuals with a chronic cough that has persisted for more than three weeks should seek professional care (not just undertake self-care). However, the cost of health services matters to many rural residents, particularly the poor ones, in China. Many studies in different developing countries show that health seeking behaviour is sensitive to the price of the services and that the poor families tend to make more use of facilities staffed by sub-professionals and have less access to care from qualified doctors [11-13]. The results from our study illustrate the magnitude of the financial burden placed on the households of TB suspects and TB patients, especially those with low income. The total annual medical expenses of TB suspects with low income accounted for over 45 percent of total annual household income. Even for high income households, the costs amounted to over 16 percent of income. We found an even greater financial burden for the diagnosed TB patients in our study.

Meng and colleagues [14] reported a heavy financial burden for 190 new smear-positive TB patients in a study in four rural counties of Shandong Province in China. The Shandong study also found that some vulnerable groups, such as the elderly, the less educated, women and those living far from health facilities faced great difficulties in reaching TB services [15]. A social assessment of a TB project funded in Inner Mongolia by the World Bank and DFID revealed similar problems of high financial burden placed on TB suspects and TB patients in the areas where the DOTS programme was implemented [16].

Xu and colleagues reported TB patients' expenditure on TB care and transportation/accommodation in 2 relatively affluent rural areas of China: one with and one without the DOTS programme [17]. All newly diagnosed and registered TB patients in these 2 counties in 2002 were interviewed and followed up for the 6-month period of their treatment. There was unexpectedly high mean TB-related expenditure in both counties, even in the one supposedly offering subsidised care. The financial burden was particularly heavy for the poorest tenth of the TB patients, who spent, on average, the equivalent of more than the whole annual household income on TB medical expenditure. The authors concluded that the revised DOTS programme did not substantially remove the financial barriers to care; it merely shifted the barriers from expenditure after diagnosis to expenditure before diagnosis. The results suggested that the providers were finding ways of maintaining their income by generating additional diagnostic and other charges for patients [17]. An earlier paper from the same survey found longer total diagnostic delays in the DOTS programme county, which supports this interpretation [3]. This is further supported by a study of 334 smear-positive patients in three rural counties in 2000/01, analysing three different models of TB care delivery [18]. The study found that for all three models, control and case management approaches were, to some extent, adapted to generate maximum income to the providers. This drive for income led to fewer cases detected, administration of unnecessary procedures and drugs, and a higher than necessary cost to the patients. In line with the results of Xu and colleagues [17], the authors of the latter study found that the vast majority of TB patients paid for their medical expenditure out-of-pocket and the total average costs to patients were about equal to the annual per capita income of the counties [18].

All these findings raise a very serious question. Has free TB diagnosis and treatment introduced through the DOTS programme really removed a financial barrier in seeking TB care and improved equity in access to and use of TB services? Under the DOTS programme, smear positive or severe smear negative TB patients are provided with drugs free of charge. Nevertheless, the cost of anti-TB drugs only accounted for a small proportion of total costs of TB diagnosis and treatment. In order to generate more revenue for their institution and themselves, TB dispensaries often give TB patients other drugs, such as liver protection drugs, unnecessary diagnostic tests, in-patient stays and even extend treatment for more than the standard 6 months, as reported by Zhan and colleagues [18]. This indicates that equity in relation to TB care remains a serious issue in tackling the TB epidemic in China. Without a radical change in financing of health services in general and of TB services in particular, the future success of TB control in China is in jeopardy.

### Quality of the implemented DOTS programme

Several commentators have concluded that the implementation of the DOTS programme in China has contributed to a great reduction in TB prevalence [1,9]. There is also an assumption that China could continue this reduction in prevalence by expanding the coverage of the DOTS programme. Others have questioned the extent to which the implementation of DOTS programme contributed to the reduction of TB prevalence [2]. The national TB control programme has set a target of reaching 100 percent coverage by the DOTS programme throughout China and it seems that this could be achieved. Nevertheless, it is the *quality *of DOTS implementation, rather than its coverage, that may be more important for the reduction of TB prevalence.

One typical example illustrates this point. According to the regulations, as long as a TB diagnosis laboratory has been set up at a county TB dispensary, that county has met one of five components of the DOTS strategy, irrespective of whether the diagnostic tests are subsequently carried out. Our findings show that only 9.4% of TB suspects surveyed had one or more sputum tests. This may imply that the poor implementation of DOTS programme failed to help increase TB case detection rates in these areas where huge financial and human resources have been invested.

In an average rural county of China, only the county TB dispensary has a facility to conduct sputum tests. When TB suspects go to county general hospitals or township health centres, doctors there are likely to offer them an X-ray examination, if they suspect that their clients are possible TB cases. This is reflected in the findings from our study: a higher proportion (27%) of TB suspects reported having an X-ray examination than a sputum test. X-ray examination is more widely available in Chinese health facilities than a sputum test. It is also quicker and more expensive (the latter would help generate revenue for service providers). Hence, X-ray examination is commonly preferred by Chinese doctors for TB diagnosis. Even so, only slightly more than one quarter of TB suspects had X-ray examinations when they sought care, which is far from satisfactory.

As a whole, the quality of DOTS implementation in most rural areas of China has not been as good as the China's national TB policy-makers and the international community have assumed. China may well reach the target of 100 percent DOTS geographic coverage by the end of 2005, but how the DOTS programme can reach all sections of the population with appropriate, affordable treatment, including the poor who have the highest prevalence, is a formidable challenge facing the national TB control programme in China.

## Conclusion

Though the DOTS achieved good performance in China overall in the last decade, our findings from the latest national random sample survey showed that access to appropriate, affordable TB services is still problematic in some rural areas of China, and receipt of care and affordability declines with declining socio-economic position.

The low income groups, the less educated people and farmers are high risk groups for TB. However, they have been marginalized by the formal professional health care system in rural China. Financial difficulty emerges as the most critical barrier to access and affordability.

These findings highlight the current shortcomings of the national TB control programme in China and the formidable challenge it faces if it is to reach all sections of the population, including the poor with the highest prevalence. The government has more to do to ensure the access of the poor to TB-related services, even in the areas where the DOTS strategy has been fully implemented.

## Competing interests

The author(s) declare that they have no competing interests.

## Authors' contributions

TZ, ST and MMW were responsible for the conception and design of the study. TZ and GJ performed the data analysis. All authors participated in interpretation of the findings. TZ, ST and MMW drafted the manuscript. GJ and ST revised and commented on the draft and all authors read and approved the final version of the paper

All authors confirm that the content has not been published elsewhere and does not overlap or duplicate their published work.

## Pre-publication history

The pre-publication history for this paper can be accessed here:



## References

[B1] China Tuberculosis Control Collaboration (2004). The effect of tuberculosis control in China. Lancet.

[B2] Squire SB, Tang S (2004). How much of China's success in tuberculosis control is really due to DOTS?. Lancet.

[B3] Xu B, Jiang Q-W, Xiu Y, Diwan V (2005). Diagnostic delays in access to tuberculosis care in counties with and without the national Tuberculosis Control Programme in rural China. Int J Tuberc Lung Dis.

[B4] Fei Y, Wang J, Zhang J, Gu Q (2006). Access to tuberculosis care among community patients with chronic cough in Yangzhong County, Jiangsu Province. Wei Sheng Yan Jiu.

[B5] Ministry of Health (MoH) (2004). General Report of the 3rd National Health Service Survey in China.

[B6] Armitage P, Colton T, eds (1998). Encyclopedia of Biostatistics.

[B7] Dahlgren G, Whitehead M (2007). A framework for assessing health systems from the public's perspective: the ALPS approach. International Journal of Health Services.

[B8] Tang S, Squire B (2005). What lessons can be drawn from TB control in China in the 1990s? An analysis from a health system perspective. Health Policy.

[B9] Chen X, Zhao F, Duanmu H, Wan L, Wang L, Du X, Chin D (2003). The DOTS strategy in China: results and lessons after 10 years. Bull World Health Org.

[B10] Gao J, Tang S, Tolhurst R, Rao K (2001). Changing access to health services in urban China: implications for equity. Health Policy and Planning.

[B11] Berman P (1986). Cost analysis as a management tool for improving the efficiency of primary care: some examples from Java. Int J Health Plann Manage.

[B12] Duggal R, Amin S (1989). Cost of health care: a household survey in an Indian district, Bombay.

[B13] Gertler P, van der Gaag J (1990). The willingness to pay for medical care: evidence from two developing countries.

[B14] Meng Q, Li R, Cheng G, Blas E (2004). Provision and financial burden of TB services in a financially decentralised system: a case study from Shandong, China. Int J Health Plann Manage.

[B15] Cheng G, Tolhurst R, Li R, Meng Q, Tang S (2005). Factors affecting delays in the tuberculosis diagnosis in rural China: a case study in four counties in Shandong Province. Trans R Soc Trop Med Hyg.

[B16] Zhang T, Liu X, Bromley H, Tang S Perceptions of tuberculosis and health seeking behaviour in rural Inner Mongolia, China. Health Policy.

[B17] Xu B, Dong H, Zhao Q, Bogg L (2006). DOTS in China – removing barriers or moving barriers?. Health Policy & Planning.

[B18] Zhan S, Wang L, Yin A, Blas E (2004). Revenue-driven in TB control – three cases in China. Int J Health PLann Mgmt.

